# Evaluating the impact of labor induction on autism spectrum disorder risk

**DOI:** 10.1007/s00404-025-08160-x

**Published:** 2025-08-26

**Authors:** Omri Zamstein, Tamar Wainstock, Eyal Sheiner

**Affiliations:** 1 The Obstetrics and Gynecology Division, Soroka University Medical Center, Ben-Gurion University of the Negev, P.O.B 151, Beer-Sheva, Israel; 2 School of Public Health, Faculty of Health Sciences, Ben-Gurion University of the Negev, Beer-Sheva, Israel

**Keywords:** Labor induction, Autism spectrum disorder, Perinatal exposure

## Abstract

**Purpose:**

Significant effort has been made in recent years to identify environmental factors—particularly perinatal exposures—that contribute to the development of autism spectrum disorder (ASD), yet many proposed associations remain inconsistent and inconclusive. Given the common use of labor induction for both medical indications and maternal preference, we aimed to investigate its potential association with ASD development, while accounting for synergistic factors that may influence its onset.

**Methods:**

A population-based cohort study was conducted at a tertiary referral center, focusing on singleton births. The study aimed to compare the occurrence of ASD in children, considering both hospital and community-based diagnoses, in relation to whether labor was induced (using mechanical cervical ripening or prostaglandins, with or without oxytocin) or began spontaneously. A Kaplan-Meier survival curve was employed to assess the cumulative incidence of ASD, and a Cox proportional hazards model was used to account for confounding variables.

**Results:**

Among 115,081 births, 13,071 (11.4%) were labor induced, with the remainder beginning spontaneously. Pregnancy complications, such as gestational diabetes mellitus, preeclampsia or eclampsia, and non-reassuring fetal heart rate patterns, were significantly more common in the labor induction group (*p*<0.001 for all). During follow-up, 767 children were diagnosed with ASD: 1.0% in the labor induction group and 0.6% in the spontaneous labor onset group (*p*<0.001). The Kaplan-Meier analysis showed a significantly higher cumulative hazard for ASD diagnosis in the labor induction group (log-rank *p*-value <0.001). However, after adjusting for maternal and perinatal factors such as maternal age, cesarean delivery, ethnicity, and gestational conditions, no significant association was found between labor induction and ASD risk (adjusted HR = 1.21, 95% CI 0.99–1.47, *p* = 0.063).

**Conclusion:**

Labor induction was associated with a higher ASD incidence but not as an independent risk factor after adjusting for maternal and perinatal factors.

## What does this study add to the clinical work

**Table:** 

This large population-based study found that labor induction was associated with a higher incidence of autism spectrum disorder (ASD) in unadjusted analyses but was not independently associated with ASD after adjustment. These findings support the continued use of current labor induction practices without modification based on ASD concerns.	

## Introduction

Autism spectrum disorder (ASD) is a complex neurodevelopmental condition with multifactorial origins, where environmental influences interact with genetic predispositions to shape its development [1]. The process of brain development in utero is exceptionally intricate and vulnerable [2], making it a focal point for understanding how environmental factors, particularly perinatal exposures, contribute to ASD. Advanced maternal age, infections acquired during pregnancy, diabetes mellitus, hypertensive disorders, preterm birth, and low birth weight have all been linked to an increased risk of ASD in offspring. However, the overall picture remains incomplete, with many suggested associations lacking consistency [3–7].

The fetal brain undergoes significant growth and development until the moment of birth [2]. Spontaneous labor onset under normal physiological conditions reflects neurohormonal and psychological readiness for extrauterine life [8]. However, certain maternal and fetal conditions often require proactive labor initiation, whether at preterm, early-term, or full-term gestational ages [9–11]. Labor induction—a widely used intervention—employs mechanical cervical ripening, prostaglandin administration, and oxytocin augmentation to achieve delivery [12–14]. While these methods are generally considered safe and effective [15], they may subtly alter the intrauterine environment and interact with fetal development during the final stages of pregnancy [16].

Previous studies exploring the association between labor induction and neurodevelopmental outcomes, including ASD, have yielded mixed results. Some investigations suggest a potential link between labor induction and an increased risk of ASD [17], while others find no such association or attribute observed links to confounding factors such as maternal comorbidities or perinatal complications [18, 19]. A recently reaffirmed committee opinion by the American College of Obstetricians and Gynecologists advises against changes in the current practices of labor induction and augmentation solely to mitigate ASD risk [20]. Potential mechanisms hypothesized to underlie a connection between labor induction and ASD include hormonal, immunological, and inflammatory pathways [21, 22], which may be influenced by the induction process.

With the increasing use of labor induction for both medical indications and patient preferences [23–25], it is essential to clarify its implications for offspring health [26]. This study aims to investigate the relationship between labor induction and ASD, considering the complex interplay of factors that influence ASD onset.

## Materials and methods

This retrospective cohort study, conducted at Soroka University Medical Center (SUMC) from 2005 to 2021, examined the association between the mode of labor initiation and the likelihood of ASD diagnoses in offspring. The study population comprised deliveries of patients insured by Clalit Health Services (CHS), the largest health maintenance organization in Israel. Given that CHS provides universally accessible healthcare, and its insured population reflects the general demographics of southern Israel [27], this study offers a comprehensive reflection of the region’s population.

The analysis compared two groups of pregnancies: those in which labor was induced and those in which labor began spontaneously, serving as the comparison group. In our practice, labor induction for patients with an unfavorable cervix is most commonly achieved using a single- or double-balloon catheter, with locally applied prostaglandins, primarily prostaglandin E2, used less often. Oxytocin is generally administered after cervical ripening to augment labor progression. Patients presenting in spontaneous labor may also require oxytocin for augmentation, though its use in these cases is less common [28, 29].

**Fig. 1:**
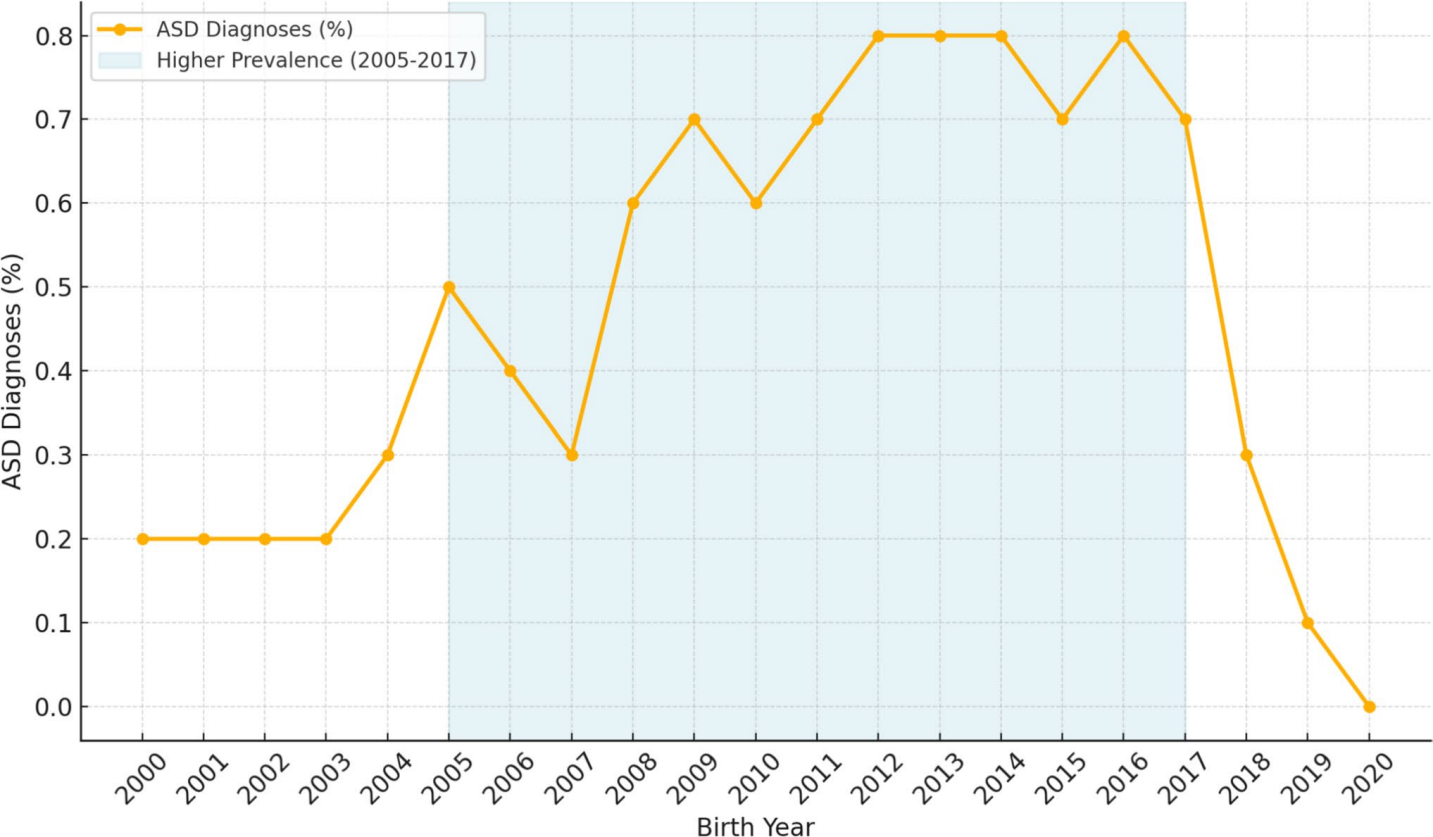
Autism spectrum disorder diagnoses (ASD; %) per birth year (years 2000–2020), showing relatively higher prevalence between the years 2005 and 2017

Obstetrical and perinatal outcomes were extracted from the SUMC Perinatal Database, which contains information recorded shortly after delivery by attending obstetricians and verified by medical secretaries. ASD diagnoses in offspring during childhood were evaluated based on CHS outpatient clinic records and hospital records. Diagnoses were made by qualified professionals, including developmental pediatricians, pediatric neurologists, child psychiatrists, and clinical psychologists, based on DSM-5 criteria [30]. These criteria require persistent deficits in social communication and interaction, along with restricted and repetitive patterns of behavior, interests, or activities, identified through developmental history and direct behavioral observation. The CHS database is routinely updated and widely used in epidemiological research, with previous studies confirming its accuracy and reliability in capturing clinical diagnoses [31]. To strengthen the analysis, the study focused on births occurring between 2005 and 2017. This timeframe was chosen to account for the lower awareness of ASD prior to 2005 and to exclude offspring born after 2017, who might not have been diagnosed by 2021 [32, 33] (Fig. 1). Pregnancies involving significant congenital anomalies or higher-order multiples were excluded from the analysis.

**Fig. 2:**
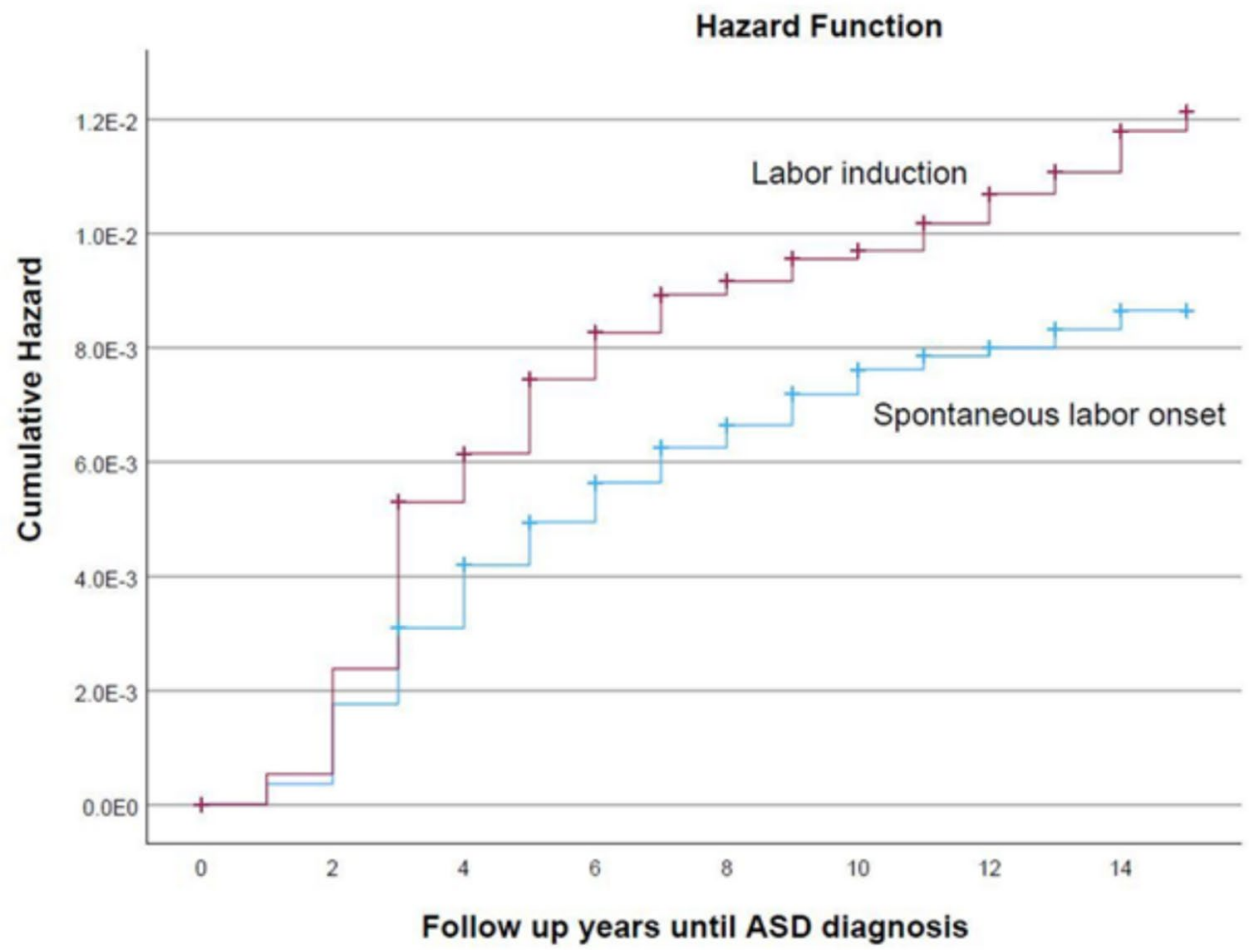
Cumulative autism spectrum disorder (ASD) incidence: comparison between induced and spontaneous labor (log-rank *p*-value < 0.001)

Ethical approval for this study was obtained from the Institutional Review Board of Soroka University Medical Center (IRB: 0357-19-SOR).

### Statistical analyses

Statistical analysis was performed using SPSS for Windows (Version 29.0; IBM, Chicago, IL). Categorical variables were presented as frequencies, while normally distributed continuous variables were expressed as means with standard deviations. Group differences were assessed using the Chi-square test for categorical variables and the unpaired t-test for continuous variables. Cases involving perinatal mortality were excluded from long-term analyses. Kaplan–Meier survival curves were generated to evaluate the cumulative incidence of ASD diagnoses during childhood, and the Cox–Mantel Log-rank test was used to identify differences between the groups. Cox regression analysis was conducted to compare morbidity while adjusting for statistically and clinically relevant confounders. All analyses were two-sided, with a p-value of < 0.05 considered statistically significant.

## Results

Among 115,081 births, 13,071 (11.4%) involved labor induction, while the remainder began spontaneously. Pregnancy complications, including gestational diabetes mellitus (9.0% vs. 3.4%), preeclampsia or eclampsia (8.1% vs. 2.7%), fetal growth restriction (FGR; 4.6% vs. 1.7%), and non-reassuring fetal heart rate patterns (10.3% vs. 5.8%), were significantly more common in the labor induction group (*p*<0.001 for all; Table 1). Conversely, preterm deliveries were more frequent in the spontaneous labor onset group (7.1% vs. 5.3%, *p*<0.001).

**Table 1:** Comparison of maternal and obstetric characteristics between labor induction and spontaneous labor onset

Characteristic	Labor induction (*n* = 13071, 11.4%)	Spontaneous labor onset (*n* = 102,010, 88.6%)	*p*-value
Maternal age (mean ± SD)	28.1 ± 5.7	28.2 ± 5.7	0.006
Primiparity (%)	34.1	23.7	<0.001
Ethnicity (%) Bedouin Jewish	55.1 44.9	61.8 38.2	<0.001
Maternal diabetes mellitus (%)	9.0	3.4	<0.001
Preeclampsia or eclampsia (%)	8.1	2.7	<0.001
Mean gestational age (weeks ± SD)	39.4 ± 2.1	38.9 ± 1.9	<0.001
Preterm delivery (<37 weeks, %)	5.3	7.1	<0.001
FGR^a^ (%)	4.6	1.7	<0.001
Non-reassuring fetal heart rate patterns (%)	10.3	5.8	<0.001
Prolonged second stage of labor (%)	1.6	0.8	<0.001
Cesarean delivery (%)	13.4	15.4	<0.001
5-min Apgar < 7 (%)	0.5	0.7	0.016
Cord blood pH < 7.0 (%)	0.7	0.3	0.068
Birthweight, gr. (mean ± SD)	3,201± 577	3,183 ± 506	<0.001
Birth weight < 2500 grams (%)	8.7	7.1	<0.001
Offspring gender Female (% all) Male (% all)	49.2 50.8	49.4 50.6	0.7
Perinatal mortality (cases, %)	299 (2.3)	581 (0.6)	<0.001

^a^Fetal growth restriction

**Table 2:** Autism spectrum disorder (ASD) risk in relation to labor induction and spontaneous labor onset

Characteristic	Labor induction (*n* = 13,071, 11.4%)	Spontaneous labor onset (*n* = 102,010, 88.6%)	*p*-value
ASD (cases, %)	125 (1.0)	642 (0.6)	<0.001
Adjusted HR^a^	1.21 (95% CI 0.99–1.47)	1 (*reference*)	0.063

^a^Adjusted for maternal age, gender, preterm birth, cesarean delivery, ethnicity, preeclampsia or eclampsia, gestational age, maternal age, gestational diabetes mellitus, epidural analgesia, and non-reassuring fetal heart rate tracing

In terms of delivery outcomes, the labor induction group experienced a higher incidence of prolonged second stage labor yet had a slightly lower cesarean delivery (CD) rate compared to the spontaneous labor group (13.4% vs. 15.4%, *p*<0.001). Birth weight was marginally higher in the induction group, while low birth weight infants (<2500 g) were more common in the spontaneous labor group (8.7% vs. 7.1%, *p*<0.001).

During follow-up, 767 children were diagnosed with ASD, with a higher incidence in the labor induction group compared to the spontaneous labor onset group (1.0% vs. 0.6%, *p*<0.001). Kaplan–Meier analysis revealed a significantly higher cumulative hazard for ASD diagnosis in the labor induction group (log-rank *p*-value <0.001; Fig. 2). However, after adjusting for maternal, delivery, and fetal factors, such as maternal age, mode of delivery, ethnicity, and gestational conditions, the Cox proportional hazards model found no significant association between labor induction and ASD risk (adjusted HR = 1.21, 95% CI 0.99–1.47, *p* = 0.063; Table 2).

## Discussion

In this cohort study of more than 115,000 singleton pregnancies, labor induction was linked to increased rates of pregnancy complications, such as gestational diabetes, preeclampsia, and FGR. It was also linked to a lower CD rate and higher birth weights. While a slight increase in the incidence of ASD was noted among offspring of induced labors, adjustment for maternal and perinatal factors revealed no evidence of an independent association between labor induction and ASD risk. These findings suggest that while labor induction may be linked to certain obstetric risks, it does not directly contribute to ASD development.

The labor induction rate in our cohort was lower than that reported in other high-income countries [9], likely reflecting population characteristics such as higher parity and, in certain segments of the population, inadequate or delayed prenatal care, which can limit timely identification and management of conditions that might otherwise lead to induction. Consistent with most major studies investigating a potential link between labor induction and the development of ASD in offspring [18–20], our study also found no evidence that labor induction is associated with an increased risk of ASD. A proposed link between labor induction and ASD focuses on endogenous oxytocin’s role in regulating social behavior and cognitive function [34]. Dysregulated oxytocin pathways have been linked to atypical behavioral patterns, prompting the hypothesis that excessive oxytocin administration during induced labor may disrupt fetal brain receptor activity and contribute to ASD [35]. Furthermore, induced labor is associated with a skewed hormonal and inflammatory profile [36, 37], which could theoretically alter the in-utero environment and impact fetal neurodevelopment. However, these hypotheses remain unsupported by clinical evidence [38]. It is therefore unlikely that a transient supra-physiologic exposure to synthetic oxytocin—which crosses the placenta in relatively small amounts [39] and must also traverse the fetal blood-brain barrier to exert any effect—could result in long-term neurological sequelae. Medications other than oxytocin, including pain management medications commonly used during induced labor, could theoretically impact fetal development. However, studies have not identified any significant risks associated with these medications, and their safety profiles during labor are well-established [40–42].

Observational studies have identified numerous other maternal, obstetric, and perinatal factors potentially associated with ASD development. These include pregnancy complications such as diabetes mellitus and hypertension, intrapartum events that may impair fetal oxygenation, maternal age and gestational age at birth [43–46]. However, the precise role of these factors in ASD development remains uncertain, as no single factor has been definitively identified as causative. Instead, they are thought to interact with pre-existing genetic susceptibilities, potentially amplifying the risk of ASD [47]. The labor induction rate in our cohort, approximately 11%, is notably lower than the 20-30% reported in developed countries [24]. This discrepancy may be partially attributed to the higher gravidity within our population, as individuals with prior births typically require fewer interventions compared to those experiencing their first labor [48]. Furthermore, certain segments of our patient population exhibit lower adherence to recommended prenatal care [49], potentially leading to fewer diagnoses of medical conditions that might otherwise necessitate earlier delivery. Unsurprisingly, induced labors were associated with higher rates of hypertensive disorders, diabetes mellitus, and FGR, likely serving as the underlying indications for labor induction [11]. Additionally, a twofold increase in abnormal fetal monitoring during and around labor was observed, which in itself can serve as an indication for labor induction. Regarding labor progression, although induced labors were characterized by slower progression, the likelihood of achieving vaginal delivery was either unaffected or improved, as evidenced by the reduced CD rates in the induction group.

Although our study did not find an independent association between labor induction and ASD, further research is needed to explore potential subgroup differences. Specifically, future studies should assess whether ASD risk varies based on specific induction methods, oxytocin dosages, or maternal comorbidities. Additionally, long-term follow-up studies should investigate broader neurodevelopmental outcomes beyond ASD, including cognitive, behavioral, and social functioning.

Our study has several limitations inherent to its observational design, which prevents establishing or refuting causation. Labor induction was classified based on the clinical decision to initiate induction, without accounting for the specific method used or the actual course of labor. Patient-level data on induction techniques (e.g., mechanical vs. pharmacological), oxytocin exposure (dose and duration), and other intrapartum medications were unavailable, preventing assessment of whether certain strategies or exposure levels might influence ASD risk. The comparatively low induction rate may reduce applicability to settings with higher rates. Additionally, population-specific factors, such as lower adherence to care, may delay access to medical services and affect the accuracy of ASD diagnoses, particularly given variations in diagnostic criteria over time. The study’s primary strengths include its large sample size, high follow-up rates, and strong generalizability. This is largely due to the comprehensive, accessible, and free medical care provided to all individuals under the National Health Insurance Law, ensuring equitable healthcare access regardless of socioeconomic status [50].

Labor induction is a widely used obstetric intervention with well-established clinical benefits [9]. In our study, we found no evidence of an independent association between labor induction and ASD in children. Given that labor induction now accounts for nearly one-quarter of births in high-income countries, including for non-medical indications [51], these findings suggest that current labor induction practices should not be modified based on concerns about ASD risk. Nonetheless, its safety and potential long-term effects on both the mother and offspring warrant continued investigation.

## Data Availability

The data supporting this study’s findings are not publicly available due to institutional and ethical restrictions but may be shared by the corresponding author upon reasonable request and subject to approval.

## Declarations

### Presentation

This study was presented in part at the 2025 SMFM Pregnancy Meeting, January 27–February 1, 2025, Aurora, Colorado, USA.
